# Two distinct modes of ocular drift observed during figure-ground perception

**DOI:** 10.1007/s00221-026-07320-y

**Published:** 2026-07-23

**Authors:** August Romeo, Maria Solé Puig, Hans Supèr

**Affiliations:** 1 Department of Cognition, Development and Educational Psychology, Faculty of Psychology, University of Barcelona, Pg. Valld´Hebron 171, 08035 Barcelona, Spain; 2 Institute of Neurosciences of the University of Barcelona (UBNeuro), Barcelona, Spain; 3 Pediatric Research Institute Hospital Sant Joan de Déu, Barcelona, Spain; 4 Catalan Institution for Research and Advanced Studies (ICREA), Barcelona, Spain

**Keywords:** Fixational eye movement, Eye synchrony, Micro saccade, Perception

## Abstract

Precise coordination between neurons and cortical areas is crucial for sensory perception, yet in vision, continuous fixational eye movements constantly shift retinal input. Traditionally considered random, these drifts modulate neural activity and may challenge the temporal precision needed for perceptual integration, particularly given their partial interocular independence. Recent evidence suggests that ocular drifts are not purely stochastic and play an active role in visual processing. Here, we investigated the impact of fixational eye movements on visual perception using a figure–ground segmentation task. Participants maintained central fixation while viewing textures composed of oriented line segments and reported the presence or absence of a centrally embedded figure. We simultaneously recorded binocular drift and microsaccades, analyzing interocular coordination through 2D cross-correlograms of angular velocities. We found that figure-presents trials exhibited narrow peaks in interocular correlation, while figure-absent trials showed broader, less synchronized drift patterns. Microsaccades were observed to facilitate drift alignment across the eyes. The decrease in microsaccade rate in figure-present trials may contribute to the change in drift synchrony, but it is not required for it. Changes in eye synchrony are also observed under monocular viewing conditions, whereas variations in accommodation and pupil size appear to be unrelated. These results identify two distinct ocular drift modes associated with different perceptual outcomes. Our findings suggest that ocular drift operates under closed-loop control and contributes to effective visual processing.

## Introduction

Precise spatiotemporal coordination between neurons and cortical areas is essential for the perceptual processing of sensory stimuli. In vision, the sensory organs, the retinas of both eyes, are in continuous motion due to eye movements, causing the spatial position of sensory input on the retina to change constantly. During gaze fixation, when perceptual processing takes place, the eyes drift slowly with occasional small rapid jerks (microsaccades). Ocular drift is considered to be random motion (Engbert et al 2011; Herrmann et al 2017; Ben-Shushan et al. 2022). As it modulates neural activity in the visual brain, it poses a challenge to the precision of spatiotemporal interactions between neurons in the processing of sensory stimuli. This is even more so because ocular drift directions are partially uncorrelated in both eyes (Ivanchenko et al. 2018).

Neural activity from the central circuitry within the oculomotor system is the source of drift (Ben-Shushan et al 2022). Increasing evidence indicates that ocular drift is binocular and constitutes a controlled form of eye motion that plays a critical role in visual processing (Rucci & Desbordes 2003; Rucci & Victor 2015; Intoy et al. 2020; Khademi et al. 2024). Ocular drifts exhibit selectivity for specific image patterns (Malevich et al. 2020), are task dependent (Friedman & Komogortsev 2025), have a role in foveal crowding (Clark et al. 2024; Prahalad et al. 2025), and cannot be attributed to convergence responses, pupillary constrictions, or head movements (Malevich et al. 2020; Pijpaert et al. 2025). In addition, drift behaviour affects visual acuity (Clark et al. 2022), reading (Bowers and Poletti 2017) and fine motor control (Ko et al. 2010).

Visual perception relies on correlations in neural activity magnitudes (Buzsáki 2006). Synchronization among neural assemblies has been identified as a fundamental mechanism for the integration of sensory information (Eckhorn et al. 1988; Singer 1999). Temporal correlations in feature detection, mediated by amplitude modulation of neural responses to stimuli, are considered a key binding mechanism (Engel & Singer 2001; Fries 2009). Figure–ground segmentation, the organization of sensory features into coherent perceptual objects, emerges from recurrent interactions between cortical areas, which are themselves contingent on the degree of synchronization within the visual cortex. Feedforward signals in visual areas are modulated by top-down feedback to segment figure from ground (Lamme and Roelfsema 2000). For the feedback to be effective, the firing of these neurons must transition from loosely synchronized (i.e., broad peak correlation curve) to a more precise synchronization regime (i.e., thin peak correlation curve; Supèr et al. 2003; van der Togt et al 2006). These states represent receptive and attentive states, respectively. A variety of factors, including sub-cortical neuro-modulatory systems, thalamocortical and cortical feedback, and sensory input influence cortical state.

To investigate the potential influence of fixational eye movements on visual perception, we recorded drifts and microsaccades while participants performed a textured figure–ground discrimination task, reporting the presence or absence of a centrally presented figure. The textures comprised line segments whose orientations differed between figure and background. To quantify interocular similarities in ocular drift, we computed two-dimensional cross-correlograms of angular velocities. During central fixation, narrow correlation peaks emerged when a figure was present, whereas absent figures were associated with broader peaks. Microsaccades facilitated alignment of drift direction between the two eyes. These findings reveal two distinct modes of ocular drift during sensory processing and support a role for closed-loop control mechanisms in visual perception.

## Materials and methods

### Participants

The study was approved by the Ethics committee of the Faculty of Psychology of the University of Barcelona in accordance with the ethical standards laid down in the 1954 Declaration of Helsinki. We obtained written informed consent from all participants before participating in the experiments. Seventeen participants took part in the figure-ground experiment (3 men and 14 women, 21.47 ± 2.76 age). The data were previously used in an earlier study (Solé Puig et al. 2021), which demonstrated that this sample size is sufficient to yield statistically robust effects. All participants were naïve to the purpose of the study and had normal or corrected-to-normal vision. Participants received credit points for taking part in the experiment.

### Apparatus

We used EventIDE software (Okazolab Ltd, London, UK) for presenting the stimuli. The display resolution was 24 pixels per degree (size 1024 × 768 pixels). The participants' position of gaze was monitored using a head-mounted binocular EyeLink II eye-tracking system at 500 Hz (SR Research System, Ontario, Canada). In addition, we used a chinrest to avoid head movements.

### Procedure

Participants sat in a dimly lit room, at 47 cm from the PC. The eye tracking equipment was calibrated (binocular; 9 points) for each participant at the beginning of the experiment.

### Figure detection task

**Fig. 1:**
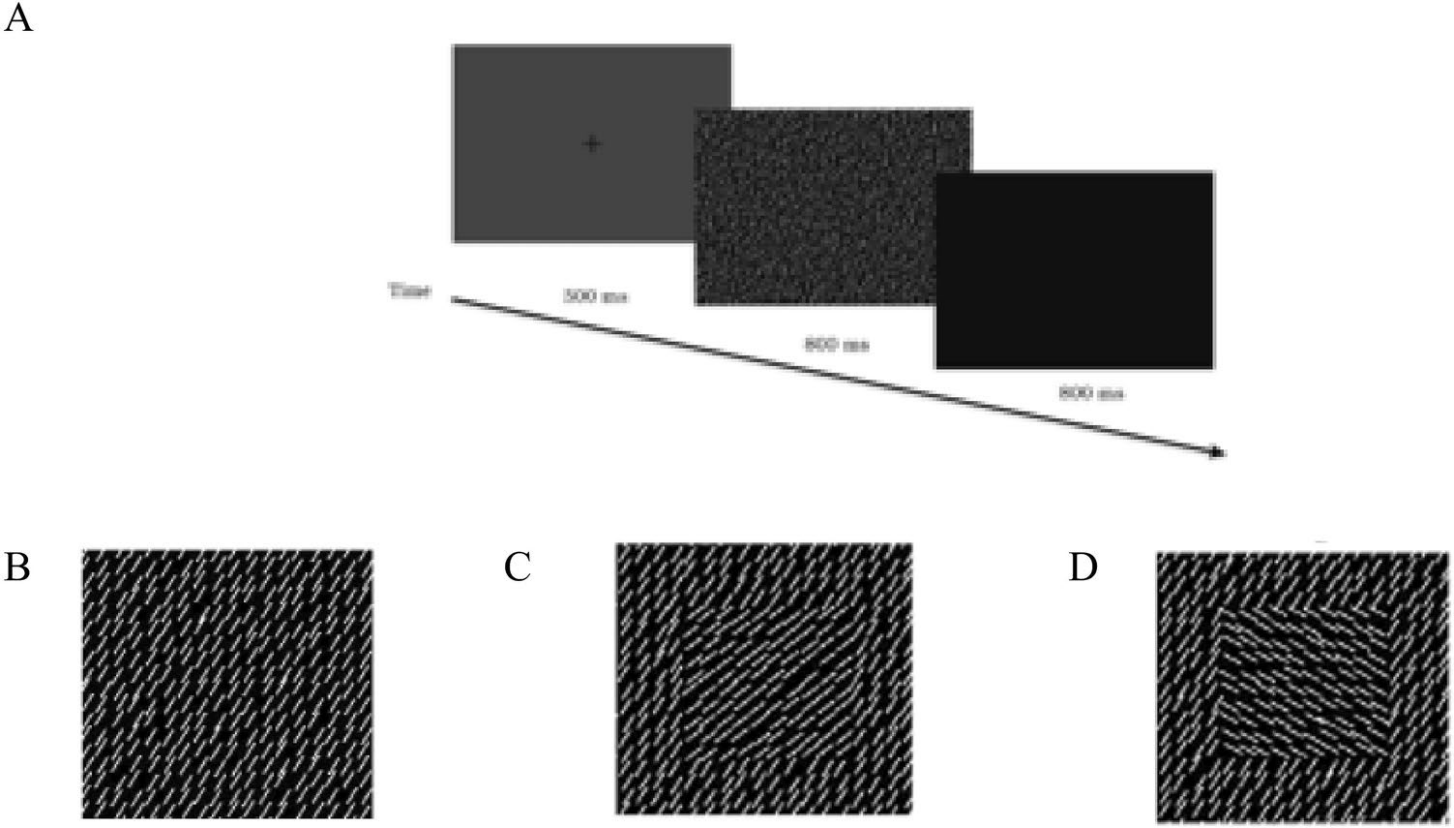
Description of the figure-ground task. **A** Each trial started with a mask containing a central fixation cross displayed for 500 ms, followed by a figure-ground stimulus for 800 ms. Afterwards, a ‘blank’ inter-trial mask was presented for 800 ms. Throughout the task subjects had to maintain central fixation and respond when a figure was present. B-D: Examples of a figure-absent stimulus (**B**) and a figure-present stimulus with a 20° (**C**), and a 90° (**D**) orientation difference

Observers were required to fixate a black central cross (size of 1.45° × 1.45°) on a grey background (Fig. 1). After 500 ms fixation, an orientation-defined figure-ground texture was presented for 800 ms. In the central region (size of 6.0° × 6.0°), line segments (size of 0.95° × 0.1°) had a 0°, 20° or 90° difference in orientation compared to the line orientation in the surrounding region. The 20° or 90° conditions were used to modify the perceptual saliency of the figure. Central line orientation was 45°, 65° or 135° when surrounding lines had a 45° orientation, and 135°, 155° or 225° when the surrounding lines had a 135° orientation. We used the MATLAB (The MathWorks Inc.) “patch” function to create the line segments.

Trials with a difference in line orientation between figure and surrounding regions were labelled as figure-present trials whereas homogeneous textures (i.e., with a 0° difference between the orientation of the central lines and surrounding segments) were labelled as figure-absent trials. Figure-present and figure-absent trials were randomly interleaved, and participants had to respond by pressing the space bar as fast and accurately as possible to indicate whether they detected a figure. If no figure was seen, subjects should refrain from responding. After the stimulus, a black inter-trial mask was presented for 800 ms and the next trial started automatically. Feedback was not given to the observers. For every subject there were 16 trials per stimulus type, which made a total of 48 trials (3 studied types × 16 trials/type).

### Control tasks

We performed two controls experiments. In both tasks, figure trials with a 90° difference in line orientation between the figure region and surrounding were used. Fifty percent of these trials contained just a homogeneous texture and were randomly interleaved with the rest. Participants viewed the figure-ground stimuli with one eye (monocular) or with both eyes (binocular). A black carton board was placed in front of one eye preventing the stimulus from being seen by that eye, but without blocking the eye tracking. Three blocks were conducted (binocular, right eye and left eye) with 120 trials in each block. In a second control task, participants were instructed to focus on a LED placed 2 cm in front at the centre of the screen (= “near” condition) instead of the central cross. In another block, the LED was switched off during the presentation of the cross and participants re-focused on the screen plane (= “screen” condition). Twelve different participants performed these control tasks.

### Calculation ocular drift correlation

**Fig. 2:**
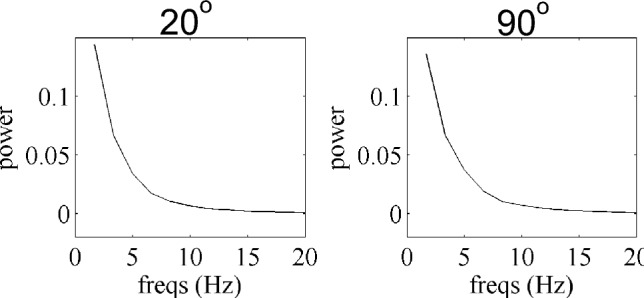
Power analysis results for the different figure-present conditions (20°, 90°). The shown lines refer to only data from post-stimulus period

Power spectrum analysis confirms that ocular drifts are primarily composed of low frequency components below 3 Hz (Fig. 2). Therefore, a low-pass filter (2.5 Hz) was applied to extract relevant frequencies. Before calculating eye synchrony, trials containing blinks or large saccadic eye movements were removed. In total, 19% of trials were excluded. Blinks were detected by samples with a negative value, which is given by the eye tracker software when the eyes are not found. Large saccades were spotted using a velocity threshold (see e.g. Engbert & Kliegl 2003 and refs therein). Then time derivatives were determined to evaluate the properties of eye coordinate velocities in the plane where tracker images are formed. The applied expressions can be set as.

Cross-correlation R for *t*_L_, *t*_R_

$$ \Re \left( {t_{L} ,t_{R} } \right) = \left\langle {\hat{v}_{L} ,\left( {t_{L} } \right) \cdot \hat{v}_{R} ,\left( {t_{R} } \right)} \right\rangle = \left\langle {\cos \Psi \left( {t_{L} ,t_{R} } \right)} \right\rangle $$

$$ \hat{v}_{L/R} \left( t \right) = \frac{{\vec{v}_{L/R} \left( t \right)}}{{\left\| {\vec{v}_{L/R} \left( t \right)} \right\|}} $$

The present vectors come from the normalization.

where these velocities are obtained as

$$ \left\{ {\begin{array}{*{20}l} {\vec{v}_{L} \left( t \right) =_{dt}^{d} \vec{L}\left( t \right),\vec{L}\left( t \right) = \left( {L_{x} \left( t \right),L_{y} \left( t \right)} \right)} \hfill \\ {\vec{v}_{R} \left( t \right) =_{dt}^{d} \vec{R}\left( t \right),\vec{R}\left( t \right) = \left( {R_{x} \left( t \right),R_{y} \left( t \right)} \right)} \hfill \\ \end{array} } \right. $$

R(*t*_L_, *t*_R_) is the averaged cosine of the angle formed by the velocities of the left eye and of the right eye in the image plane, $\overrightarrow {{\mathbf{v}}} L$, $\overrightarrow {{\mathbf{v}}}_{R}$, taken in general at different times *t*_L_, *t*_R_. This magnitude is measuring the strength of the correlations between the two velocity directions. For zero lag, the cosine is close to + 1/− 1 when the two eyes are moving in the same/opposed direction. Adopting the time lag definition τ = *t*_L_ − *t*_R,_ averaged cross-correlation functions *c*(τ) can be found from the pre-stimulus and from the late post-stimulus periods. Such averages are symbolically given by $c\left( \tau \right) = \frac{1}{{t_{f} - t_{i} - \tau }}\int\limits_{{t_{\tau } + \tau }}^{{t_{f} }} {dt\Re \left( {t,t - \tau } \right)} ,$ where *t*_*i*_, *t*_*f*_ are the limits of the considered period (in practice the integration is calculated as a discrete sum). All correlations curves for the different *t* values contributing to the averaged correlation [c(τ)] were obtained and the width and height for every curve determined. For studying the degree of eye motion synchrony from the shape of these functions we used the width at one third of the peak height (the same criterion as van der Togt et al 2006) as our measure of ‘peak width’. This produces a distribution of widths, which can be statistically tested.

### Microsaccade detection

The times when microsaccades occur are determined applying the criterion explained by Engbert-Kliegl 2003 (see also McCamy et al. 2015). We take approximations to the time derivatives of the eye coordinates. Next, we consider the instantaneous deviations of these velocities from their mean values, say Δ*v*(*t*), and compare the magnitude of that deviation and the standard deviation of the velocity itself *σ*_v_. The start of a microsaccade is the time *t* when $(\Delta v(t))^{2} > (1 + \lambda^{2} )\sigma_{v}^{2}$. In our case *λ* = 4 was set.

On some occasions the time course of the coordinates of one eye can qualify as a microsaccade (while the movement of the other just involves variations ‘under the threshold’). These ‘monocular’ events are relatively scarce, but nonetheless present (11.2% of the considered set). This issue may relate to threshold values, as some studies have reported the absence of monocular saccades (Fang et al. 2018). For truly binocular microsaccades, the overlap in time appears to be quite large, as initiation times for the left and for the right eye are quite close to each other. When they do not fully coincide, differences of one or very few time sample(s) are detected [our time sample length was 2 ms]. For 97.8% of the considered cases there was coincidence within 6 ms. Of these, anti-conjugate movements with microsaccade-like velocity are possible. To estimate their occurrence, we counted trials where cosΨ stayed near –1 (–0.99 to –1) for ≥ 10 samples. This occurred in 9.8% of figure-present trials.

### Statistical analysis

For the analysis of the transition in synchrony we compared the averaged correlations curve over the pre-stimulus period (400 ms before stimulus onset to stimulus onset) to the late post-stimulus interval (from 400 to 800 ms after stimulus onset. For statistical analysis of the microsaccade rates we used t-tests.

## Results

### Behavior responses

Participants performed a figure-ground detection task (Fig. 1). The average success rates were 87.70% for 20° trials and 98.18% for 90° trials, and the behavioral reaction times (mean ± std) 529 ± 86 ms for 20° trials and 486 ± 84 ms for 90° trials. Performance was thus high for the 90° condition and slightly lower in the 20° condition.

### Ocular drift

**Fig. 3:**
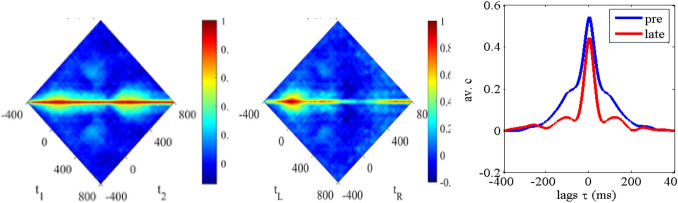
2D auto-correlograms (**A**), 2D cross-correlograms (**B**) and average cross-correlation curves (C) for figure-present trials in the 90°condition

To evaluate individual drift pattern over time, we performed an auto-corelation analysis of the angular eye velocities for each eye separately. The 2D auto-correlograms from the figure-present trials reveal periods with abroad correlation peak during pre and late post stimulus periods (Fig. 3A). Changes in the width of the correlation peaks likely reflect changes in duration over which the eye maintains movement in a particular direction. Accordingly, the results suggest that the eyes drift for extended periods in a single direction.

But do both eyes drift in the same direction? To address this, we computed 2D cross-correlograms of angular eye velocities from both eyes (Fig. 3B). The cross-correlation curves (Fig. 3C) revealed a reduction in peak width during the late post-stimulus period. For the 20° condition, the mean peak width decreased from 92.6 ms (pre-stimulus) to 66.0 ms (late post-stimulus; rank-sum test, *p* < 10⁻⁶), and for the 90° condition, from 112.3 ms to 44.7 ms (*p* < 10⁻⁶). Thus, correlations between the eyes diminish after stimulus onset, but around 400 ms post-stimulus, drift directions begin to re-synchronize. The observed patterns in the average cross-correlation are more likely to arise from the eyes moving in the same direction at the same time, rather than from differences in temporal lag. Accordingly, narrow peaks indicate that both eyes move in the same direction for shorter durations than during the pre-stimulus period.

### Figure-ground

**Fig. 4:**
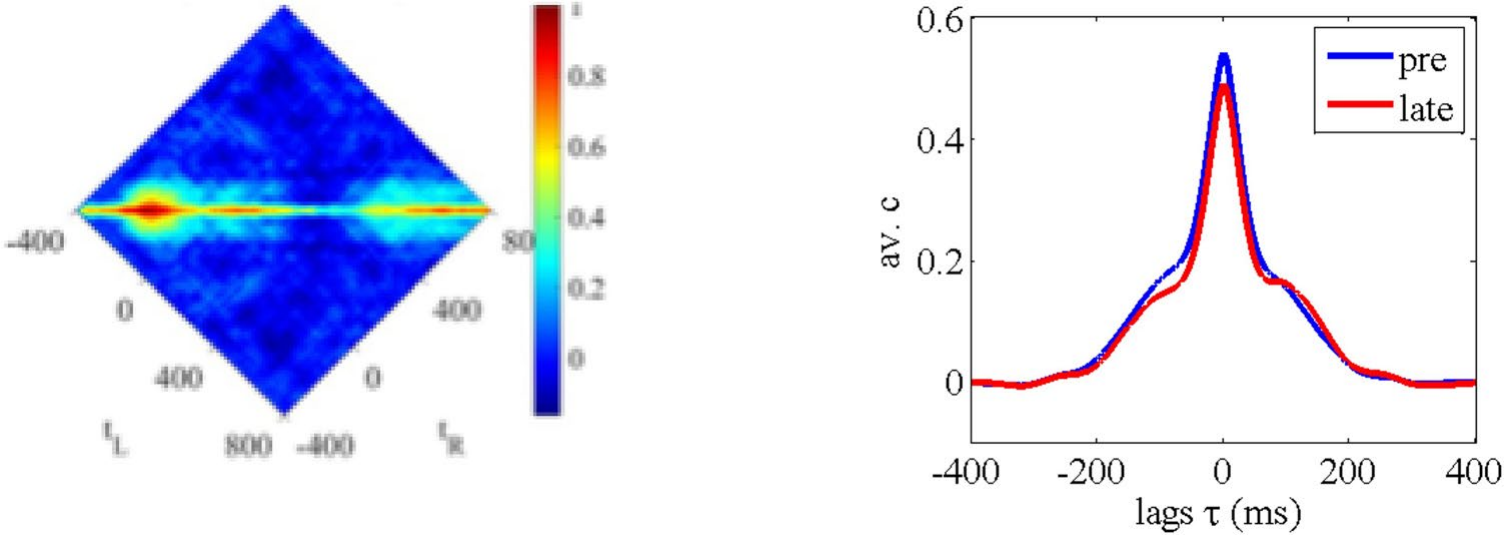
2D cross-correlograms (**A**) and average cross-correlation curves (**B**) for figure-absent trials

To determine whether the narrowing of the correlation peak depends on stimulus configuration, we analysed the 2D cross-correlograms from figure-absent trials (Fig. 4A). In the figure-absent trials, the average cross-correlation curves (Fig. 4B) remained similar between the pre-stimulus and late stimulus periods. In fact, a slight increase in peak width was observed from 93.5 ms to 110.5 ms (*p* ≈ 2 × 10⁻^4^). These results indicate that the alignment of ocular drift direction is dependent on the configuration of the stimulus.

### Binocular vs monocular viewing

**Fig. 5:**
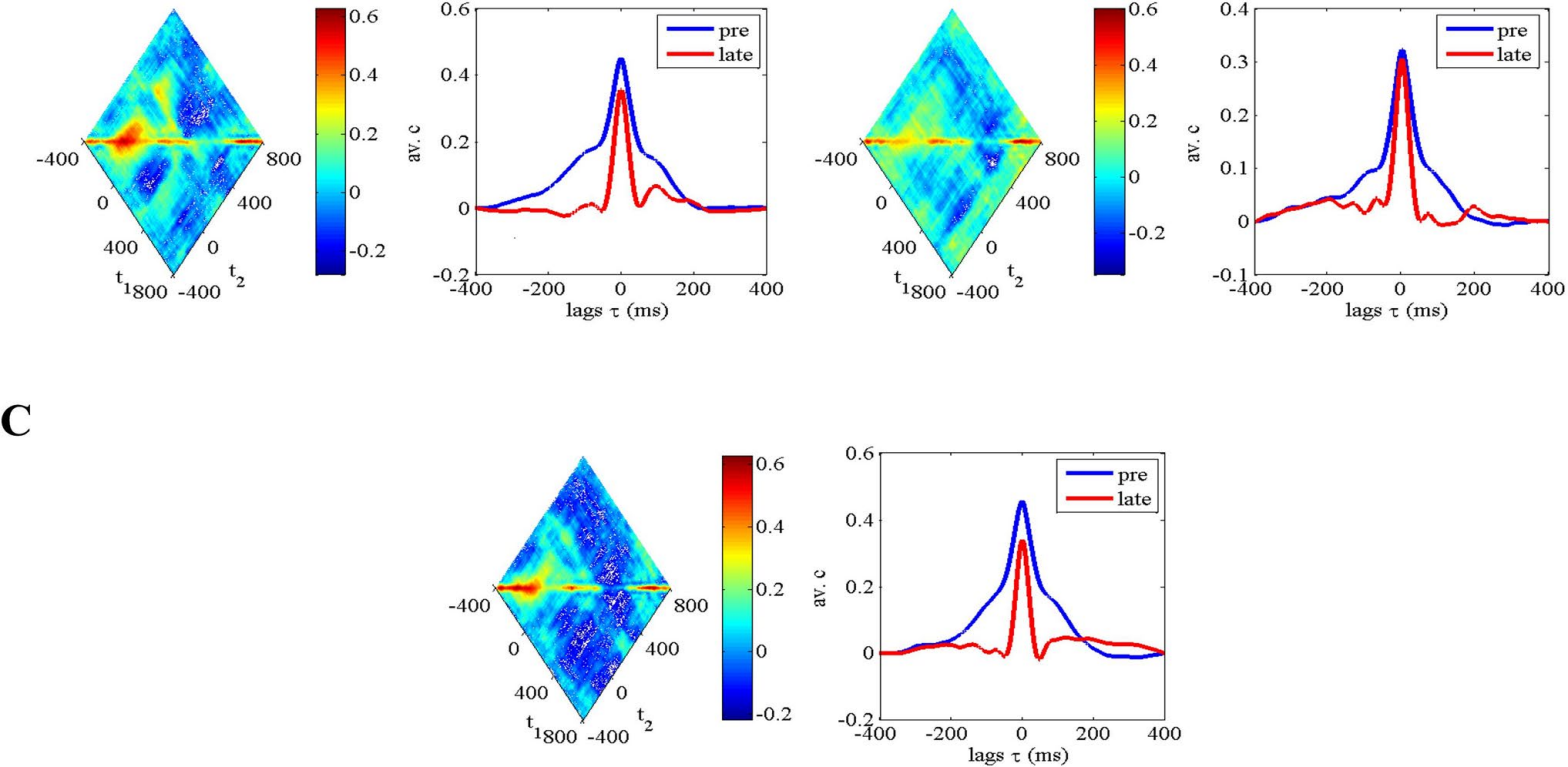
Cross-correlograms and mean correlation curves from the control experiment involving the monocular left (**A**) and right (**B**) conditions, and the binocular condition (**C**)

Any disparity in the two retinal images is a depth cue and can be used by visual system to produce a dis-conjungate (vergence) eye movement. To eliminate binocular disparity as a potential confound, we tested monocular viewing. The average detection performances for figures were 97.3 ± 7.2%, 93.8 ± 11.8% and 98.0 ± 4.4% for binocular, right eye and left eye conditions, respectively. Reaction times were 484 ± 93 ms (binocular), 484 ± 87 ms (right eye) and 487 ± 96 ms (left eye). The change in velocity alignment synchrony can be seen in monocular viewing conditions as well. In the set of trials when the figure is seen, the results of the monocular condition (Fig. 5A,B) resemble the ones of the binocular condition (Fig. 5C).

For monocular, left eye, the mean peak width undergoes a change when comparing pre-image and late image stages. Its value decreases from 115.2 to 32.0 ms (p < 10^–6^) when the figure is seen. For monocular, right eye (dominant eye), the mean peak width falls from 78.7 to 32.0 ms (p < 10^–6^). In the binocular condition, there is a narrowing from 106.7 to 27.5 ms (p < 10^–6^).

### Accommodation and vergence

**Fig. 6:**
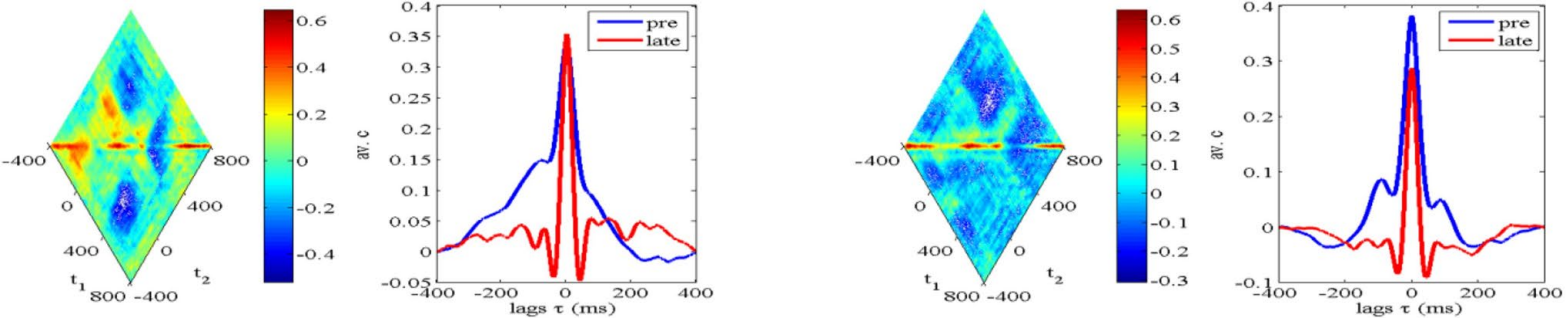
Cross-correlograms and mean correlation curves from the control experiment involving the ‘far’ (**A**) and the ‘near’ (**B**) conditions

In a second control task, we assessed possible involvement of accommodation and vergence. Vergence, the movement of both eyes in opposite directions and is therefore a disconjugate (asynchronous) eye movement, has a role of figure-ground perception (Solé Puig, et al. 2021). To evaluate the potential effect of accommodation and vergence, participants changed focus in the second control task. The average detection performances and behavioural reaction times of the figure textures were 97.2 ± 6.1% and 490 ± 90 ms, and 90.1 ± 15.8% and 520 ± 80 ms for “screen” and “near” conditions, respectively. For “near” condition, the mean peak width undergoes a change when comparing pre-image and late image stages (Fig. 6A). Its value decreases from 86 to 24 ms (p < 10^–6^) when the figure is seen. For “far” condition (Fig. 6B), the mean peak width decreases from 55 to 22 ms (p < 10^–6^). The high detection performance indicates that accommodation changes likely did not produce blur, but we cannot rule out that they affected oculomotor behaviour.

### Pupil

**Fig. 7:**
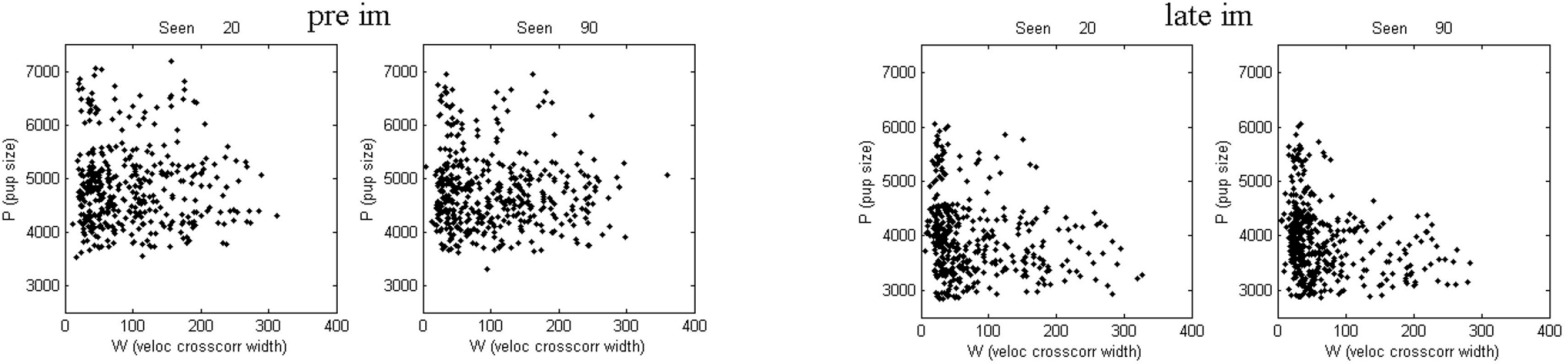
Scatter plots for synchrony vs pupil size per trial. One for pre-stimulus (‘pre-im’) period and another to post (‘late im’) stimulus period. The adopted synchrony measure is the velocity cross-correlation peak width value, indicated by W

Pupil responses interact with vergence responses and accommodation as part of the so-called near-triad. To assess a possible influence of pupil responses, we compared pupil size with velocity alignment synchrony. In line with previous findings (Malevich et al. 2020), we didn’t find correlation between pupil size and ocular drift synchrony (Fig. 7).

**Fig. 8:**
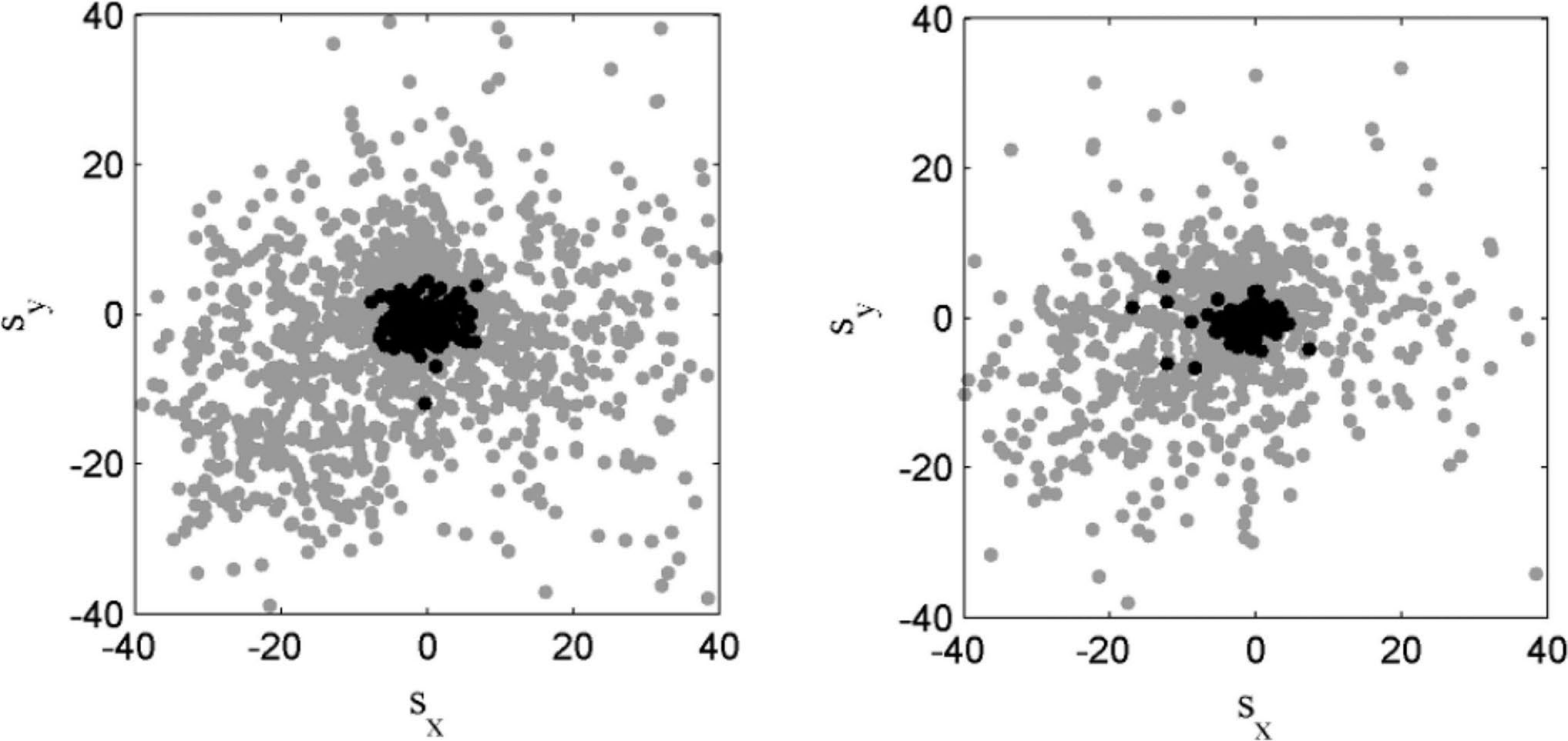
Average gaze position during the first 800 ms in figure-present and figure-absent trials. Gray points represent gaze position samples from single trials across all subjects. Black dots indicate the average positions across subjects

In addition, we examined potential biases in the eye data and found no evidence of systematic bias in gaze position. Previous analyses of microsaccade direction relative to the visual stimulus (i.e., line segment orientation) also revealed no bias (Fig. 8).

### Microsaccades

**Fig. 9:**
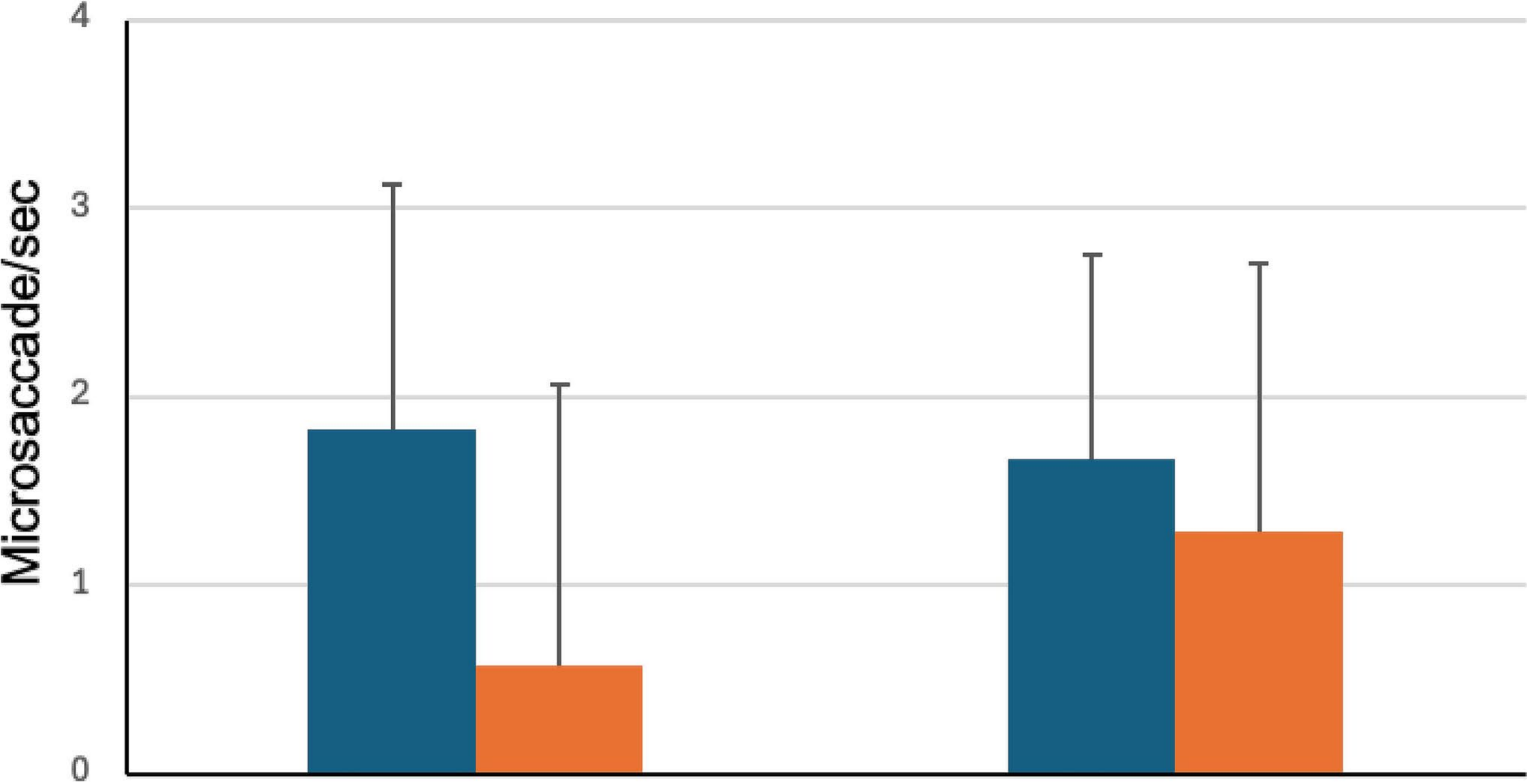
Microsaccade rates in pre-stimulus (blue) and post-stimulus (orange) periods

The synchronization of ocular drift direction may be triggered by microsaccades, given that they are binocular events. To investigate this, we first analysed microsaccade rates (Fig. 9). In figure-present trials, microsaccade rates dropped by approximately 68% after stimulus onset (pre-stimulus: 1.82 ± 1.3 Hz; post-stimulus: 0.57 ± 1.08 Hz; *p* = 2.0 × 10^−46^). In contrast, figure-absent trials showed a smaller reduction of 24%. Notably, post-stimulus microsaccade rates were significantly higher in figure-absent trials (1.28 ± 1.43 Hz; *p* = 0.006), while pre-stimulus rates did not differ between conditions (1.67 ± 1.49 Hz; *p* = 0.97; Fig. 9). These findings indicate that a significant drop in microsaccade rate occurs only when a figure is present. The fact that microsaccade rates decrease around the time of a task has been shown before, e.g. in Pastukhov and Braun 2010.

**Fig. 10:**
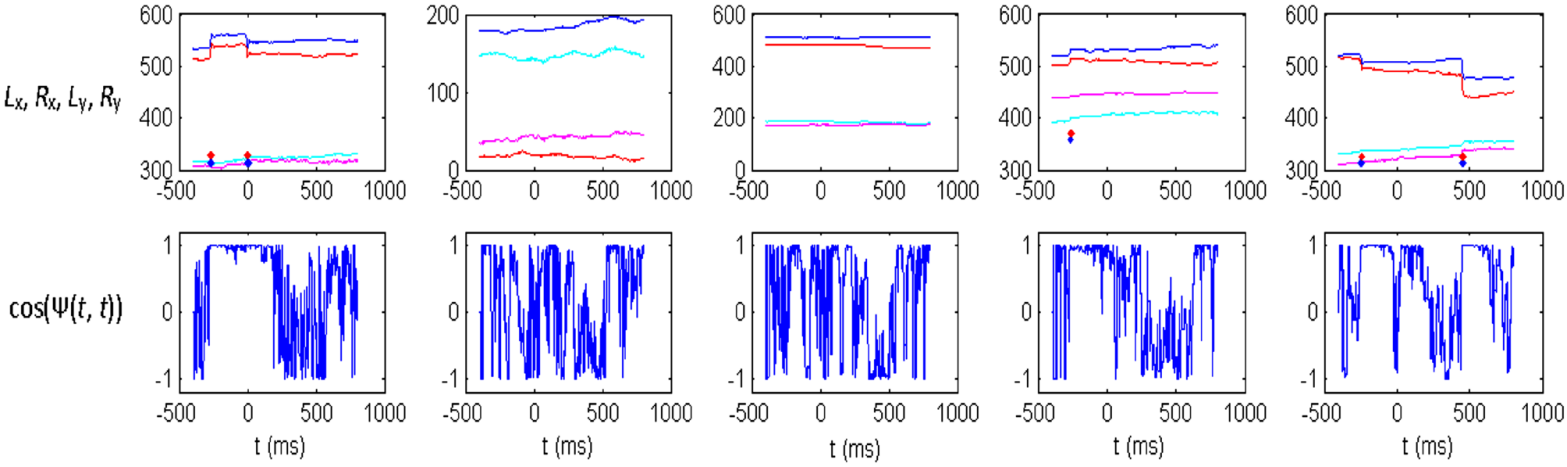
Eye position (raw data) and velocities (smoothed data) for zero lag. Time courses of the recorded L_x_, R_x_, L_y_, R_y_ eye coordinates in blue, red, cyan and magenta, respectively (top row), measured in eye tracker pixels, and of the cosine of the angle between the resulting left eye and right eye velocities cos(Ψ(t, t)) (bottom row), for five randomly selected trials. The times of the detected microsaccades are the abscissa values of the superimposed dots. As can be seen, cos(Ψ(t, t)) ≈ 1 for a period of the order of a hundred ms after every microsaccade

**Fig. 11:**
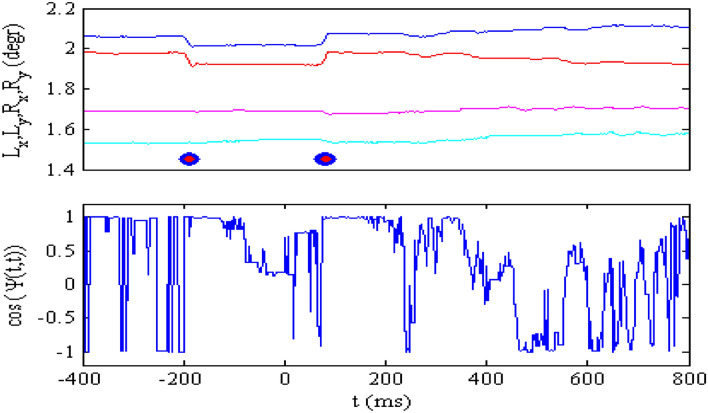
Larger scale view of a pair of microsaccades. Position traces are now given in degrees of visual angle. Comparing top and bottom parts one may appreciate that the cos(Ψ) ≈ 1 part starts when position curves are changing their slope, i.e., when velocities become visibly nonzero

To test whether microsaccades may have a role in ocular drift synchrony, we measured the duration of alignment in angular eye velocities following each microsaccade. Specifically, we calculated the time interval during which cos(Ψ(t, t)) at zero lag remained above 0.99, indicating near-perfect alignment. We found that, following a microsaccade, the velocities of both eyes are tightly aligned for a short period (Figs. 10,11). In figure-present trials, the average alignment duration was 107 ± 57 ms before stimulus onset and 111 ± 67 ms after (*p* = 0.57). In figure-absent trials, alignment duration slightly decreased from 126 ± 31 ms pre-stimulus to 110 ± 69 ms post-stimulus (*p* = 0.02). These results indicate that microsaccades transiently align drift direction between the eyes.

**Fig. 12:**
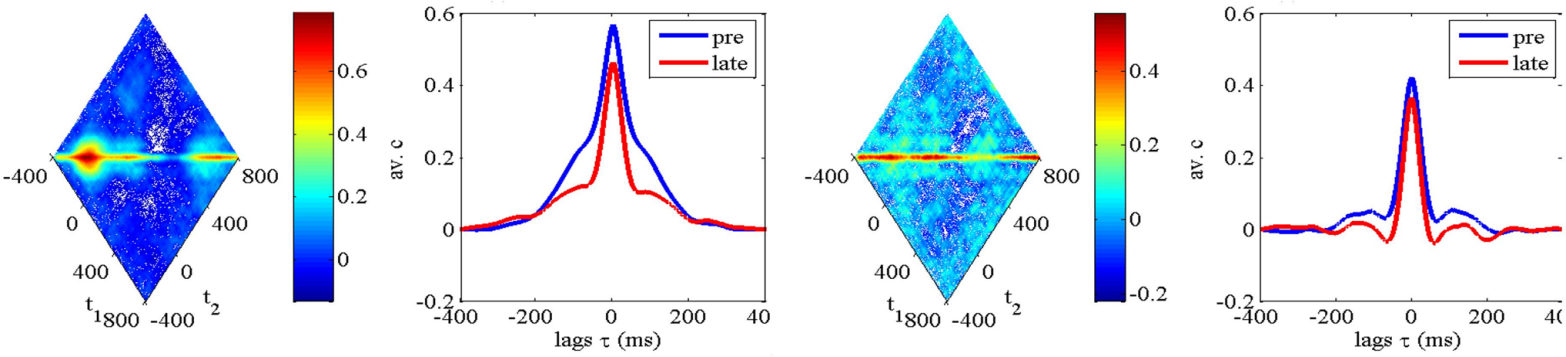
Cross-correlograms R(t_1_,t_2_) and mean cross-correlations as functions of the time lag c(τ) after merging data for 20° and 90° conditions. A and B pairs correspond to the results from trials with and without microsaccades, respectively. The effects caused by the presence of microsaccades can be appreciated

We next aanalysed the correlation curves from trials with and without microsaccades, orientations confounded (Fig. 12). In figure-present trials, when microsaccades occurred, the correlation curves exhibited broad peaks in the pre-stimulus period and much narrower peaks post-stimulus (Fig. 12A). In contrast, in trials without microsaccades, the peaks were generally narrower overall, with a modest reduction in width from pre- to post-stimulus (Fig. 12B). The change in correlation peak width was significantly larger in trials when microsaccades were present (100.2 ± 55.9 ms) compared to trials without microsaccades (38.6 ± 7.9 ms; *p* = 4.5 × 10^−36^).

## Discussion

In this paper, we described the correlation between the angular velocities, i.e., directions of ocular drift during gaze fixation of participants performing a figure-ground detection task. Ocular drift is assumed to follow a random path, like Brownian motion process or a random walk with memory (Engbert et al 2011; Herrmann et al 2017; Ben-Shushan et al. 2022). In line with previous studies (Rucci & Desbordes 2003; Rucci& Victor, 2015; Intoy et al. 2020; Malevich et al. 2020), we provide evidence for an organized oculomotor behaviour in the form of correlated direction of drift in distinct periods during visual processing.

### Microsaccades

Our findings tend to suggest that the strength of drift synchrony is modulated by microsaccades. These are binocular events that displace both eyes in the same direction. Following a microsaccade, the drift trajectories of the two eyes become aligned, and this correlation persists for a considerable period, indicating that microsaccades play a role in coordinating subsequent binocular drift dynamics. The lower microsaccade rate at post stimulus period (Pastukhov & Braun 2010) may explain the thinner correlation curve in figure-present trials. In other words, reducing or inhibiting the microsaccade rate leads to shorter periods of synchronized drift. However, they appear not to be crucial as change in drift motion and figure-ground detection occurs irrespective of the presence of microsaccades.

Microsaccades have been shown to synchronize retinal activity (Kuang et al. 2012; Masquelier et al. 2016) and modulate neural synchrony in the visual cortex (Lowet et al. 2018). Convergent, temporally correlated inputs from LGN neurons are particularly effective in driving V1 responses (Alonso et al. 1996; Usrey et al. 2000). Our findings raise the possibility that microsaccades actively shape perceptual processing by synchronizing ocular drift, thereby enhancing the temporal precision of retino–cortical interactions. Through this mechanism, microsaccades may align sensory input across the eyes, promoting coherent neural activity at early visual stages. Such motor-driven synchronization could represent a fundamental feedforward control strategy, linking eye movement dynamics to figure–ground segregation.

### Figure-ground

Feedforward signals in the visual cortex are modulated by top–down feedback to support figure–ground segmentation (Lamme and Roelfsema 2000; Supèr et al. 2001). For this feedback to be effective, neuronal firing must shift from a loosely synchronized state (broad correlation peaks) before stimulus onset to a more precisely synchronized state (narrow correlation peaks) after onset (Supèr et al. 2003; van der Togt et al. 2006). These states are commonly interpreted as “receptive” and “attentive,” respectively. Ocular drift shows a similar pattern: broad correlation curves in the pre-stimulus period and narrow peaks in figure-present trials, but not in figure-absent trials. Moreover, the pre- to post-stimulus change in eye synchrony was slightly lower in the 20° condition, which may indicate that perceptual saliency could influence eye synchrony, as reported for neural synchrony (van der Togt et al. 2006).

In the visual cortex, figure–ground signals emerge in distinct temporal windows reflecting different processing stages (Heinen et al. 2005; Corthout et al. 1999; Sugihara et al., 2011; Yamane et al., 2020). Figure–ground responses are detected around 100–150 ms (Lamme et al. 1992) and coincide with the onset of ocular convergence (Sole Puig et al., 2021). By contrast, re-synchronization of ocular drift begins around 400 ms after stimulus onset. This timing aligns more closely with attentional influences on figure–ground segmentation (Scholte et al., 2006; Koivisto & Silvanto, 2011). Thus, when the stimulus is behaviorally relevant, narrow-peak synchrony in ocular drift may reflect an attentional state, whereas in the absence of relevant stimuli the system returns to a receptive, broad-peak, desynchronized state.

### Conclusion

In line with previous studies, our findings demonstrate that fixational eye drift is not random but exhibits stimulus-dependent modulation in synchrony. The pattern suggests that eye drift reflects an interaction between oculomotor control and sensory processing, providing a functional link that may contribute to closing the visuomotor loop.

## Data Availability

All data supporting the findings of this study are available within the paper and upon request.

## References

[CR2] Alonso JM, Usrey WM, Reid RC (1996) Precisely correlated firing in cells of the lateral geniculate nucleus. Nature 383:815–819 8893005 10.1038/383815a0

[CR3] Ben-Shushan N, Shaham N, Joshua M, Burak Y (2022) Fixational drift is driven by diffusive dynamics in central neural circuitry. Nat Commun 13:1697 35361753 10.1038/s41467-022-29201-y PMC8971408

[CR5] Bowers NR, Poletti M (2017) Microsaccades during reading. PLoS ONE. 10.1371/journal.pone.0185180 28934359 10.1371/journal.pone.0185180 PMC5608362

[CR6] Buzsáki G (2006) Rhythms of the brain. Oxford Univ. Press

[CR7] Clark AM, Intoy J, Rucci M, Poletti M (2022) Eye drift during fixation predicts visual acuity. Proc Natl Acad Sci U S A 119:e2200256119. 10.1073/pnas.2200256119 36442088 10.1073/pnas.2200256119 PMC9894113

[CR8] Clark AM, Huynh A, Poletti M (2024) Oculomotor contributions to foveal crowding. J Neurosci 44(48):e0594242024. 10.1523/JNEUROSCI.0594-24.2024 39455258 10.1523/JNEUROSCI.0594-24.2024 PMC11604144

[CR9] Corthout E, Uttl B, Ziemann U, Cowey A, Hallett M (1999) Two periods of processing in the (circum)striate visual cortex as revealed by transcranial magnetic stimulation. Neuropsychologia 37:137–145 10080371 10.1016/s0028-3932(98)00088-8

[CR10] Eckhorn R, Bauer R, Jordan W, Brosch M, Kruse W, Munk M, Reitboeck H (1988) Coherent oscillations: a mechanism of feature linkingin the visual cortex? Multiple electrode and correlation analysesin the cat. Biol Cybern 60:121–130 3228555 10.1007/BF00202899

[CR11] Engbert R, Mergenthaler K (2011) An integrated model of fixational eye movements and microsaccades. PNAS 2:56. 10.1073/pnas.1102730108 10.1073/pnas.1102730108 PMC3182695 21873243

[CR12] Engbert R, Kliegl R (2003) Microsaccades uncover the orientation of covert attention. Vision Res 43:1035–1045 12676246 10.1016/s0042-6989(03)00084-1

[CR13] Engel AK, Singer W (2001) Temporal binding and the neural correlates of sensory awareness. Trends Cogn Sci 5:16–25 11164732 10.1016/s1364-6613(00)01568-0

[CR14] Fang Y, Gill C, Poletti M, Rucci M (2018) Monocular microsaccades: do they really occur? J Vision 18(3):18. 10.1167/18.3.18 10.1167/18.3.18 PMC5868759 29677334

[CR15] Friedman L, Komogortsev OV (2025) Fixation drift increases as a function of time-on-task in a brief saccade tracking study. PLoS ONE 20(6):e0310619. 10.1371/journal.pone.0310619 40471958 10.1371/journal.pone.0310619 PMC12140216

[CR16] Fries P (2009) Neuronal gamma-band synchronization as a fundamental process in cortical computation. Annu Rev Neurosci 32:209–224 19400723 10.1146/annurev.neuro.051508.135603

[CR17] Heinen K, Jolij J, Lamme VAF (2005) Figure-ground segregation requires two distinct periods of activity in V1: a transcranial magnetic stimulation study. NeuroReport 16:1483–1487 16110276 10.1097/01.wnr.0000175611.26485.c8

[CR18] Herrmann CJJ, Metzler R, Engbert R (2017) A self-avoiding walk with neural delays as a model of fixational eye movements. Sci Rep 7:12958. 10.1038/s41598-017-13489-8 29021548 10.1038/s41598-017-13489-8 PMC5636902

[CR19] Intoy J, Victor JD, Rucci M (2020) Active task-dependent control of ocular drift during natural fixation. J vis 20(11):1335. 10.1167/jov.20.11.1335

[CR20] Ivanchenko D, Hafed Z, Schaeffel D (2018) How correlated are drifts in both eyes during fixational eye movements? Invest Ophthalmol vis Sci 59:5792

[CR21] Khademi F, Zhang T, Baumann MP, Malevich T, Yu Y, Hafed ZM (2024) Visual feature tuning properties of short-latency stimulus-driven ocular position drift responses during gaze fixation. J Neurosci 44(13):e1815232024. 10.1523/JNEUROSCI.1815-23.2024 38302441 10.1523/JNEUROSCI.1815-23.2024 PMC10977026

[CR22] Ko HK, Poletti M, Rucci M (2010) Microsaccades precisely relocate gaze in a high visual acuity task. Nat Neurosci 13:1549–1553. 10.1038/nn.2663 21037583 10.1038/nn.2663 PMC3058801

[CR23] Kuang X, Poletti M, Victor JD, Rucci M (2012) Temporal encoding of spatial information during active visual fixation. Curr Biol 22:510–514 22342751 10.1016/j.cub.2012.01.050 PMC3332095

[CR24] Lamme VAF, Roelfsema PR (2000) The distinct modes of vision offered by feedforward and recurrent processing. Trends Neurosci 23:571–579 11074267 10.1016/s0166-2236(00)01657-x

[CR25] Lamme VA, Van Dijk BW, Spekreijse H (1992) Texture segregation is processed by primary visual cortex in manand monkey. Evid VEP Exp Vision Res 32:797–807 10.1016/0042-6989(92)90022-b 1604849

[CR26] Lowet E, Gips B, Roberts MJ, De Weerd P, Jensen O, van der Eerden J (2018) Microsaccade-rhythmic modulation of neural synchronization and coding within and across cortical areas V1 and V2. PLoS Biol 16(5):e2004132. 10.1371/journal.pbio.2004132 29851960 10.1371/journal.pbio.2004132 PMC5997357

[CR27] Malevich T, Buonocore A, Hafed ZM (2020) Rapid stimulus-driven modulation of slow ocular position drifts. Elife 9:e57595. 10.7554/eLife.57595 32758358 10.7554/eLife.57595 PMC7442486

[CR28] Masquelier T, Portelli G, Kornprobst P (2016) Microsaccades enable efficient synchrony-based coding in the retina: a simulation study. Sci Rep 11(6):24086. 10.1038/srep24086 10.1038/srep24086 PMC4827057 27063867

[CR29] McCamy MB, Otero-Millan J, Leigh RJ, King SA, Schneider RM, Macknik SL, Martinez-Conde S (2015) Simultaneous recordings of human microsaccades and drifts with a contemporary video eye tracker and the search coil technique. PLoS ONE 10(6):e0128428. 10.1371/journal.pone.0128428 26035820 10.1371/journal.pone.0128428 PMC4452707

[CR30] Pastukhov A, Braun J (2010) Rare but precious: microsaccades are highly informative about attentionalal location. Vision Res 50:1173–1184. 10.1016/j.visres.2010.04.007 20382176 10.1016/j.visres.2010.04.007

[CR31] Pijpaert AR, Goossens HHLMJ, van Dijk BW et al (2025) A validation study on the accuracy and precision of gaze and vergence using stereoscopic eye-tracking technology. Behav Res 57:214. 10.3758/s13428-025-02731-1 10.3758/s13428-025-02731-1 PMC12213999 40593383

[CR32] Prahalad KS, Clark AM, Moon B, Roorda A, Tiruveedhula P, Harmening W, Gutnikov A, Jenks SK, Kapisthalam S, Rucci M, Rolland JP, Poletti M (2025) Non-uniform signal pooling across the foveola. Curr Biol 35(24):6086-6099.e4. 10.1016/j.cub.2025.11.007 41338196 10.1016/j.cub.2025.11.007 PMC13244582

[CR33] Rucci M, Desbordes G (2003) Contributions of fixational eye movements to the discrimination of briefly presented stimuli. J Vis 3:852–864 14765967 10.1167/3.11.18

[CR34] Rucci M, Victor JD (2015) The unsteady eye: an information-processing stage, not a bug. Trends Neurosci 38(4):195–206. 10.1016/j.tins.2015.01.005 25698649 10.1016/j.tins.2015.01.005 PMC4385455

[CR35] Singer W (1999) Neuronal synchrony: a versatile code of the definition of relations? Neuron 24:49–65 10677026 10.1016/s0896-6273(00)80821-1

[CR36] Solé Puig M, Romeo A, Supèr H (2021) Vergence eye movements during figure-ground perception. Conscious Cogn 92:103138 34022640 10.1016/j.concog.2021.103138

[CR37] Supèr H, Spekreijse H, Lamme VAF (2001) Two distinct modes of sensory processing observed in monkey primary visual cortex (V1). Nat Neurosci 4:304–310 11224548 10.1038/85170

[CR38] Supèr H, van der Togt C, Spekreijse H, Lamme VAF (2003) Internal state of monkey primary visual cortex (V1) predicts figure-ground perception. J Neurosci 23:3407–3414 12716948 10.1523/JNEUROSCI.23-08-03407.2003 PMC6742343

[CR39] Usrey WM, Alonso JM, Reid RC (2000) Synaptic interactions between thalamic inputs to simple cells in cat visual cortex. J Neurosci 20:5461–5467 10884329 10.1523/JNEUROSCI.20-14-05461.2000 PMC6772311

[CR40] Van der Togt C, Kalitzin S, Spekreijse H, Lamme VAF, Supèr H (2006) Synchrony dynamics in monkey V1 predict success in visual detection. Cereb Cortex 16:136–148 15843628 10.1093/cercor/bhi093

